# Applying a Model of Teamwork Processes to Emergency Medical Services

**DOI:** 10.5811/westjem.2020.7.47238

**Published:** 2020-10-19

**Authors:** William G. Fernandez, Justin K. Benzer, Martin P. Charns, James F. Burgess

**Affiliations:** * UT Health–San Antonio, Department of Emergency Medicine, San Antonio, Texas; † The University of Texas at Austin, Department of Psychiatry, Austin, Texas; ‡ Boston University School of Public Health, Department of Health Law, Boston, Massachusetts

## Abstract

**Introduction:**

Effective teamwork has been shown to optimize patient safety. However, research centered on the critical inputs, processes, and outcomes of team effectiveness in emergency medical services (EMS) has only recently begun to emerge. We conducted a theory-driven qualitative study of teamwork processes—the interdependent actions that convert inputs to outputs—by frontline EMS personnel in order to provide a model for use in EMS education and research.

**Methods:**

We purposively sampled participants from an EMS agency in Houston, TX. Full-time employees with a valid emergency medical technician license were eligible. Using semi-structured format, we queried respondents on task/team functions and enablers/obstacles of teamwork in EMS. Phone interviews were recorded and transcribed. Using a thematic analytic approach, we combined codes into candidate themes through an iterative process. Analytic memos during coding and analysis identified potential themes, which were reviewed/refined and then compared against a model of teamwork processes in emergency medicine.

**Results:**

We reached saturation once 32 respondents completed interviews. Among participants, 30 (94%) were male; the median experience was 15 years. The data demonstrated general support for the framework. Teamwork processes were clustered into four domains: planning; action; reflection; and interpersonal processes. Additionally, we identified six emergent concepts during open coding: leadership; crew familiarity; team cohesion; interpersonal trust; shared mental models; and procedural knowledge.

**Conclusion:**

In this thematic analysis, we outlined a new framework of EMS teamwork processes to describe the procedures that EMS operators employ to convert individual inputs into team performance outputs. The revised framework may be useful in both EMS education and research to empirically evaluate the key planning, action, reflection, and interpersonal processes that are critical to teamwork effectiveness in EMS.

## INTRODUCTION

Despite improvements in quality and effectiveness in emergency medical services (EMS),^1^–^2^ improving patient safety remains an important, ongoing concern.^3^ As an integral component of the healthcare system, significant work has been done in EMS to improve patient safety by adopting evidence-based approaches to care.^3^–^6^ Unfortunately, research on teamwork-based strategies to improve care in EMS has only recently started to emerge.^7^ In other areas of healthcare, interventions to improve teamwork have demonstrated reductions in medical errors in the emergency department^8^,^9^ and intensive care unit,^10^ as well as the operating room setting,^11^ primarily on building teamwork competencies, such as effective communication.^12^–^17^ Building on the current teamwork literature,^8^–^17^ we sought to apply the language of the science of teamwork to the work performed in EMS. To do this, we conducted a theory-driven qualitative study of *teamwork processes*—the interdependent actions that convert inputs to outputs (or outcomes)—by frontline EMS personnel that are associated with team effectiveness.^18^–^22^

### Conceptual Framework

We define *teamwork* as the interaction of two or more individuals to perform a given task.^19^ Teamwork is the inter-related set of team member’s thoughts, beliefs, and feelings needed for the team to function as a unit.^12^ Team members see themselves—and are seen by others—as belonging to a specific social entity within an organization.^20^ *Teamwork processes* are the cognitive, verbal, and behavioral activities directed toward organizing tasks (inputs) to achieve collective goals (outputs), and form the basis for team competencies (eg, knowledge, skills, and attitudes) that are crucial for effective healthcare team performance.^18^–^19^ One of the foundational models of teamwork is the input-process-output (IPO) model.^18^–^19^,^21^ In this model, *inputs* are the individual characteristics of employees, the available organizational resources, and the demands of the task to be done. *Processes* are the interdependent actions and behaviors that convert inputs to outputs. *Outputs* include objective outcomes such as overall team performance and mission completion, as well as less tangible outcomes such as patient and employee satisfaction.^18^–^22^

**Figure 1:**
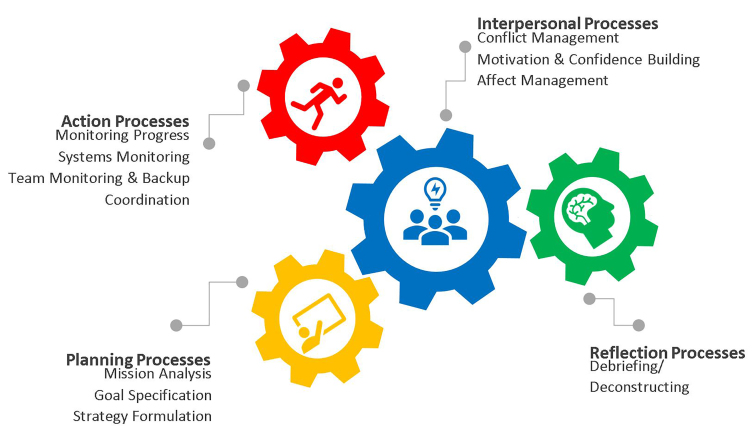
Temporal model of teamwork processes in emergency care.^18^

Building on the IPO model, Marks, Mathieu, and Zaccaro proposed a temporally based model of teamwork processes.^18^–^19^,^23^ In this framework, teamwork processes are thought to occur in interacting performance episodes: transition processes; action processes; and interpersonal processes. Further refinements to the model were proposed by Fernandez et al,^18^ who separated transition processes into planning processes (eg, setting goals and prioritizing tasks to be completed) and reflection processes (eg, feedback on areas of improvement), as these domains were thought to occur in distinct episodes of time (Figure 1).^18^–^19^,^23^ In the revised model, planning, action, and reflection processes inform one another over time, while interpersonal processes contemporaneously affect the success of the other processes.^19^,^23^ A list of teamwork processes and their definitions appear in Table 1. (See supplementary content online.)

## METHODOLOGY

### Study Design

This was a qualitative study of EMS personnel (ie, key informants) regarding teamwork in EMS. We approached individual EMS providers for enrollment via purposive sampling of personnel to complete a semi-structured, audiotape-recorded phone interview.

### Study Population

The study population was a convenience sample of fire department-based EMS agency in Houston, TX, which responds to over 225,000 911 calls annually. All firefighters in the agency have been certified at the emergency medical technician (EMT) level of training, while approximately 10% are paramedic-certified.

**Population Health Research Capsule**

What do we already know about this issue?

*Teamwork processes, critical to organizational success, may be grouped into performance episodes: planning, action, reflection, and interpersonal processes*.

What was the research question?

Can the model of teamwork processes in emergency care be extended to the EMS context?

What was the major finding of the study?

*This study provides early empirical support to applying a model of teamwork processes in emergency care to EMS*.

How does this improve population health?

*The revised model may be useful to guide future “deliberate practice” training or focused evaluation of key teamwork processes to improve teamwork performance in EMS*.

The enrollment criteria were as follows:

1. A valid state EMT license, and
2. Full-time employment in the agency.

### Study Procedures

We conducted confidential, one-on-one telephone interviews among participants to identify barriers and enablers of effective teamwork in their organization. Interviews were scheduled in advance and were conducted by calling into a conference call service (FreeConferenceCall.com, Long Beach, CA) that allowed for interviews to be recorded on a secured, password-protected site. Prior to commencing the study, we piloted interview questions with members of a separate, hospital-based EMS agency.

### Recruitment of Study Participants

Study participants were recruited through the following means: 1) recruitment email from the agency’s medical director; 2) visits to fire stations to promote the study; and 3) announcing the study at a training conference. We explained the purpose of the study, as well as identified the enrollment criteria. Those interested were contacted to set up a phone interview. We recruited participants until we achieved the point of theoretical saturation. “Theoretical saturation” occurs when additional data collection does not produce additional knowledge or understanding with respect to the study questions.^24^–^26^ In other words, this is the point at which an interviewer is able to predict the answers that participants would provide given a certain question (ie, when no new perspectives on a topic are gained).

To estimate the sample size necessary for saturation, we anticipated a baseline of 15–20 interviews.^24^–^25^ Given the degree of segmentation within the organization by professional certification (ie, paramedic vs EMT) as well as by rank (officers vs firefighters), we anticipated that we would need to sample approximately 30–40 key informants to reach theoretical saturation. Also, due to the time lag between participant enrollment and completion of phone interviews, we estimated a 50% dropout rate among enrollees. To account for this, we planned to recruit between 60–80 EMS personnel to satisfy our ultimate participation goal of 30–40 participants who would complete the telephone interview.

### Phone Interviews

Phone interviews followed a semi-structured format. Key informants were asked “grand tour” questions, that is, broad open-ended queries about the general characteristics of a given setting or role, regarding typical EMS runs during a typical shift (eg, “Can you walk me through a typical ambulance run during a typical shift?”). These “ice-breaker” questions are thought to encourage participants to feel more comfortable sharing during the interview.^26^–^27^ These were followed up with questions about specific teamwork processes (ie, planning processes – “What are you thinking/saying to your partner on the way to the scene?”; action processes – “During a typical 911 call, how are tasks divided up between partners?”; “When you’re on the way to the hospital with a patient, what sort of things are you thinking/doing?”; “Can you describe a typical interaction between the EMS crew and the hospital staff?”); reflection processes – “What sort of things happen after you’ve handed off care at the hospital and you’re on your way back to the station?”; and interpersonal processes – (eg, “How often are there disagreements about what should be done?”), routine task activities (eg, “What sort of tasks are typically required during a typical call?”), as well as task activities that required teamwork (eg, “What tasks are better done by groups of two or more, rather than by just one person?”).

Additionally, officers in the fire department were asked about supervisory/coordination activities (eg, “What makes your job managing a critical event such as a multi-casualty incident go more smoothly?”), or the role of senior leadership/management in promoting teamwork (eg, “What can senior leadership/management do to promote teamwork?”; and “How does scheduling crews for 24 hours at a time affect teamwork?”). Finally, participants were asked about enablers and barriers to teamwork in their typical work day. The complete interview protocol is available in Appendix A. The lead author conducted all interviews. No personal identifiers were included during the interviews. All interviews were audio-recorded, transcribed verbatim, and reviewed for accuracy. The institutional review board approved this study.

### Coding

We used a commercially available software program designed for qualitative data management to code data for later analysis (NVivo 11 Student Version; QSR International, Victoria, Australia). We created a codebook where the transcribed data were systematically sorted into separate, individual “chunks” of data, or codes.^26^–^27^ In this initial round of coding, the first author categorized coherent thoughts identified within the textual data using deductive, “theory-based” codes. A key part of this process was the use of “memoing” in which observations were made during the data analysis, including annotation of interesting, unique, and recurrent patterns in the text, and preliminary coding decisions were recorded. Additionally, the lead author identified inductive codes by reviewing data that was not captured within the theory-based coding; this resulted in six emergent concepts.

### Data Analysis

We used a thematic analytic approach^27^–^28^ to identify themes within the coded data. The first author conducted all data analyses by reviewing transcripts^27^ in an iterative process to engage closely with the data. Two authors combined codes into candidate themes that depicted the data accurately. Unlike codes, themes consist of ideas and descriptions that identify what the data is about and/or what it actually means.^27^ In other words, themes are distinct units of meaning that are observed in the textual data. Several candidate themes emerged from this process. Finally, all authors reviewed the candidate themes to determine how they supported the data, and how they aligned with the Marks teamwork-processes framework, as modified by Fernandez et al.^18^–^19^,^29^ All authors iteratively selected themes that were most relevant and made the most meaningful contribution to understanding what was going on within the data. The result of this deliberative process was the revised model of teamwork processes applied to EMS.

## RESULTS

**Figure 3:**
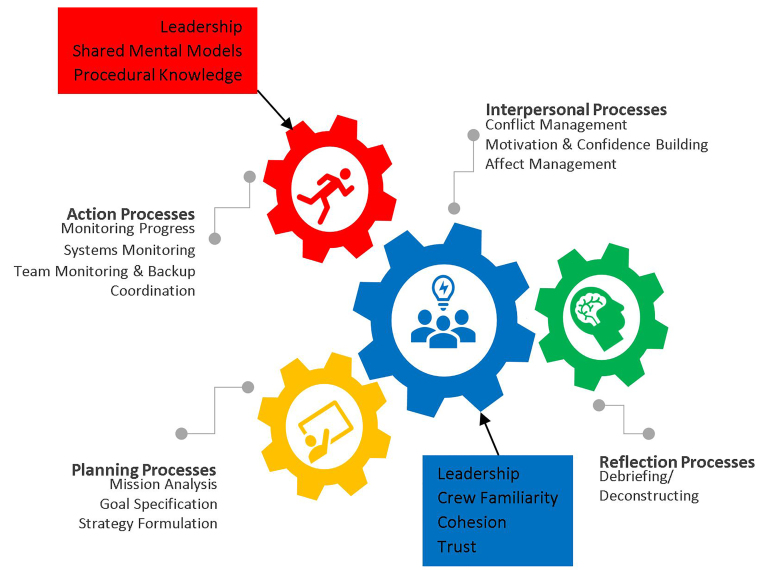
Revised model of teamwork processes in emergency care, applied to emergency medical services.

We reached a point of saturation once 32 respondents completed phone interviews. Participants were selected from across the organization, from firefighter-EMTs with one year of experience in EMS to senior fire captains with 40 years of experience; the median work experience was 15 years. The sample consisted of substantially more males than females (30 vs 2), which is consistent with the percentage in the organization as a whole. The sample consisted of substantially more paramedic-certified firefighters (28 vs 4) than those certified as EMT. The data provided general support to the existence of teamwork processes that clustered into four domains: planning; action; reflection; and interpersonal processes. Additionally, six emergent concepts were identified during the open coding phase of data analysis: leadership; crew familiarity; team cohesion; interpersonal trust; shared mental models; and procedural knowledge. A summary of themes along with illustrative quotes are presented in Table 2. (See supplementary content online.) The revised model illustrating the relationships between the emergent concepts and teamwork processes are illustrated in Figure 3.

## DISCUSSION

In this theory-driven study, we sought to apply a model of teamwork processes^18^ to EMS. Our analysis provided support to distinct teamwork processes, which were grouped into four domains: planning; action; reflection; and interpersonal processes.^18^ The data also uncovered several emergent concepts that respondents felt were central to effective teamwork in EMS: leadership^30^–^31^; crew familiarity^32^; team cohesion^32^–^33^; interpersonal trust^23^,^30^–^31^; shared mental models^34^–^25^; and procedural knowledge^36^–^37^.

Leadership was revealed as influencing both action and interpersonal processes.^30^–^31^ In other words, effective leadership is critical to ensuring that “things get done”^38^,^39^ and to creating conditions that facilitate team effectiveness.^40^ These behaviors can be broadly separated into task-focused and person-focused behaviors.^41^ Task-focused behaviors are activities that foster understanding of task requirements and the procedures for task completion.^21^,^39^,^41^ Person-focused behaviors are those that facilitate behavioral interactions, cognitive structures, and attitudes so that members can work effectively as a team.^21^,^40^,^41^ In a recent meta-analysis, both task-focused (understanding/accomplishing tasks) and person-focused behaviors (promoting norms) were important correlates of team performance.^41^ The current study shows how leadership affects EMS teamwork processes.

Additionally, shared mental models were linked to coordinated action.^34^ A study of primary care teams revealed a similar relationship, which was helpful for managing unexpected situations.^23^ Alonzo and Dunleavy^30^ showed that teammates with a shared understanding of collective tasks to be done are more likely to interpret situational cues similarly, improving coordination.^42^

Procedural knowledge, the tacit information gained from hands-on task-specific training (ie, “know-how”), was important to team monitoring and backup.^36^–^37^ Marks et al found a similar association between procedural knowledge and the development of backup behaviors through cross-training, which may improve team effectiveness.^42^

Crew familiarity was found to influence the teamwork process of affect management in our study.^32^ Crew familiarity is an aspect of team design (ie, the work schedule) that results in cohorts of individuals maintaining a stable work group over an extended period of time. Patterson et al showed that crew familiarity can influence both interpersonal and action processes.^32^ Patterson reports that EMTs work with their most frequent partner only 35% of the time.^32^ Unfamiliar EMS teams might be “unclear about their partner’s expectations and may be hesitant to speak up when necessary.”^32^ Further, unfamiliar teams are more likely to experience disruptions in team cohesion, delays in critical actions, and may threaten occupational safety among EMS crews.^31^ Additionally, Gersick noted that such unfamiliar teammates may feel “anxiety, confusion, or apprehension” as a result ofsuch lack of professional familiarity with one another.^43^,^44^ Furthermore, others noted that EMS teams with limited prior exposure to one another are more likely to experience lower quality performance.^45^–^47^

We found that team cohesion was positively related to motivation and confidence building. As noted above, the shared self-efficacy that members had when working with “my crew” gave EMS personnel a sense of collective confidence in their team’s ability to accomplish challenging tasks. Similarly, a meta-analysis showed that interpersonal attraction among teammates was associated with an increased motivation for teammates to perform well on tasks.^48^

Additionally, we found that interpersonal trust influenced conflict management. A similar relationship was observed by Benzer et al, who found that psychological safety influences the interpersonal process of conflict management.^23^ They noted that “psychological safety promotes effective interpersonal processes by strengthening a collective sense of trust,” which is closely related to the concept of trust that emerged from our interviews.^23^

Participants shared that they often compartmentalize their emotions rather than addressing them as part of open interpersonal processes. Although many EMS and fire service organizations employ psychologists, conduct occupational stress training, and sponsor in-house peer support groups, the culture within many agencies is one of “do not admit to needing help.”^48^ Similar barriers are seen in the military setting.^49^ It is presumed that the negative stereotypes reduce service members’ motivation to seek help.^50^ As in the military, normalizing the culture on seeking mental health services is necessary.^51^

This framework may be useful for EMS leaders (eg, medical directors, department chiefs, training officers) as well as researchers to identify the strengths and weaknesses in their organization’s teamwork performance during team training and evaluation. An EMS agency could then use the results of training evaluations as feedback to modify or emphasize training on weaker teamwork processes, and conversely, allocate resources away from those processes that were judged the strongest.

## LIMITATIONS

Our study had some limitations. First, we enrolled individuals at a single agency, which may limit the generalizability of our findings to other agencies. However, the respondents in this study were drawn from a range of ranks (ie, officers and firefighters) and experience levels. Second, the choice of a fire-based EMS agency may limit the generalizability of our findings to agencies whose emergency care services are not organized within a fire department structure. However, the majority of EMS agencies in the United States are fire department based.^52^ Third, we enrolled more paramedics than EMTs. However, our aim was to sample a range of EMS providers, including those in senior leadership positions. This likely led to further oversampling of paramedic-certified personnel.

## CONCLUSION

In this thematic analysis, we have outlined a model of EMS teamwork processes that describe the procedures that EMS operators employ to convert individual skills, knowledge and resources (ie, inputs) into collective team performance (ie, outputs). Although there are notable exceptions cited in this paper, the science of teamwork research in EMS is still relatively new and developing. Our findings extend prior teamwork research to the EMS context, and form the basis for an evolving model of teamwork processes in EMS. This framework of EMS teamwork processes may be useful to help EMS leaders, educators, and researchers evaluate the key processes that are critical to teamwork effectiveness in EMS. Given the relative dearth of prior attention in this area, we feel future investigation is warranted that is focused on empirically testing the utility of this model to predict outcomes based on the performance of these teamwork processes.

## Supplementary Information



## Figures and Tables

**Table 1:** Teamwork processes.^18^–^19^

Concept	Definition
Planning processes	
Mission analysis	Interpretation and evaluation of the crew’s overall mission, including the key tasks to be performed, the operating environment that will be encountered, as well as the human and material resources necessary to accomplish the pending mission
Goal specification	Identification and prioritization of goals that are aligned with, and necessary to accomplish, the overall mission
Strategy formulation	Development of contingency courses of action necessary for mission accomplishment based on current environment and available resources
Action processes	
Monitoring progress	Tracking tasks and advancement toward mission completion
Systems monitoring	Tracking team resources and external conditions
Team monitoring and backup	Awareness and anticipation of tasks to be completed, as well as assisting team members with completing a task
Coordination	Orchestrating the sequence and timing of interdependent actions
Reflection processes	
Debriefing	A critical evaluation of the events that transpired during the team’s performance
Interpersonal processes	
Conflict management	Processes that assist with interpersonal disagreements among team members
Motivation and confidence building	Processes that increase confidence and motivation among team members
Affect management	Regulating team members’ emotions to accomplish team goals

**Table 2:** Demographic characteristics of sample (n = 32).

Gender
Female: 2
Males: 30
Professional certification
EMT: 4
Paramedic: 28
Rank
Firefighter: 9
Engineer operator: 7
Captain: 12
Senior captain: 3
District chief: 1
Experience
Median: 15 years
Range: 1–40 years
