# Supplementary material for: Applying a Model of Teamwork Processes to Emergency Medical Services
Source: West J Emerg Med. 2020 Oct 19;21(6):264–71. doi: 10.5811/westjem.2020.7.47238 (PMC7673905; doi:10.5811/westjem.2020.7.47238)
Supplement: Supplementary file 1 [file wjem-21-264-s001.docx]

**Appendix A. Interview Protocol for Firefighters**

I’d like to thank you for participating in the study.

First off, just a few demographic questions:

1) PROFESSIONAL CERTIFICATION (EMT/PARAMEDIC)

2) RANK (FIREFIGHTER, EO, CAPTAIN, SENIOR CAPTAIN, ETC)

3) YEARS OF EXPERIENCE?

4) MALE/FEMALE

CAN YOU WALK ME THROUGH A TYPICAL AMBULANCE RUN DURING A TYPICAL SHIFT?

WHAT SORT OF TASKS ARE REQUIRED DURING A TYPICAL CALL?

WHAT ARE SOME OF THE THINGS THAT YOU’RE THINKING OR TALKING ABOUT WITH YOUR PARTNER ON THE WAY TO THE SCENE?

HOW OFTEN ARE YOU PAIRED UP WITH THE SAME PERSON?

HOW DOES THAT AFFECT COMMUNICATION OR TEAMWORK?

DURING A TYPICAL RUN, HOW ARE TASKS USUALLY DIVIDED UP BETWEEN YOU AND YOUR PARTNER?

CAN YOU DESCRIBE TASKS THAT ARE BETTER DONE BY TWO PEOPLE INSTEAD OF JUST ONE?

WHAT MAKES FOR AN IDEAL OR “BEST” PARTNER?

HOW OFTEN ARE THERE DISAGREEMENTS ABOUT WHAT SHOULD BE DONE?

WHEN YOU’RE ON THE WAY TO THE HOSPITAL, WHAT SORT OF THINGS ARE YOU THINKING/DOING?

CAN YOU DESCRIBE A TYPICAL INTERACTION BETWEEN THE EMS CREW AND THE HOSPITAL STAFF?

WHAT SORT OF THINGS MAKE THE HANDOFF OF PATIENT CARE GO SMOOTHLY?

CAN YOU SHARE ANY STORIES ABOUT SITUATIONS WHEN THE INTERACTION DIDN’T GO AS PLANNED?

WHAT SORT OF THINGS HAPPEN AFTER YOU’VE HANDED OFF THE PATIENT AT THE HOSPITAL & YOU’RE ON YOUR WAY BACK TO THE STATION?

IS THERE ANYTHING I’VE MISSED?

Appendix A (Cont.) Interview Protocol for Officers

I’D LIKE TO THANK YOU FOR PARTICIPATING IN THIS STUDY.

FIRST OFF, JUST A FEW DEMOGRAPHIC QUESTIONS:

1) PROFESSIONAL CERTIFICATION (EMT vs. PARAMEDIC)

2) RANK (FIREFIGHTER, EO, CAPTAIN, SENIOR CAPTAIN, ETC)

3) YEARS OF EXPERIENCE?

4) MALE/FEMALE

I UNDERSTAND THAT OFFICERS LIKE YOURSELF ARE RESPONSIBLE FOR RUNNING THE FIRE STATION…. IS THAT RIGHT?

WHAT IS THE ROLE OF THE CAPTAIN (OR EMS SUPERVISOR) IN THE FIRE DEPARTMENT?

I’M TOLD THAT FIRE CAPTAINS GET CALLED TO RESPOND UNDER CERTAIN SPECIFIC SITUATIONS. CAN YOU WALK ME THROUGH A TYPICAL SITUATION WHEN A FIRE CAPTAIN WOULD BE CALLED TO RESPOND?

WHAT TRIGGERS A CALL FOR A CAPTAIN (OR EMS SUPERVISOR, ETC)?

WHAT SORT OF TASKS ARE REQUIRED OF A CAPTAIN DURING A MEDICAL CASE?

WHAT ARE SOME OF THE THINGS THAT YOU’RE THINKING ABOUT ON THE WAY TO THE SCENE OF A CRITICAL EVENT?

HOW DO TASKS DURING A CRITICAL EVENT USUALLY GET DIVIDED UP?

IN TERMS OF SERIOUS MEDICAL CALLS (E.G., MULTI-CAR COLLISION), CAN YOU DESCRIBE TASKS THAT ARE BETTER DONE BY MORE THAN ONE PERSON INSTEAD OF JUST ONE?

IN TERMS OF FIREFIGHTING, WHAT SORT OF TASKS ARE BEST DONE BY MORE THAN ONE PERSON?

IN YOUR ROLE, WHAT MAKES YOUR JOB MANAGING A CRITICAL EVENT (E.G., MULTI CAR COLLISION, PUTTING OUT A FIRE, ETC) GO MORE SMOOTHLY?

HOW IMPORTANT IS COORDINATION ON THE SCENE OF A CRITICAL EVENT?

HOW DO YOU GET DIFFERENT PERSONNEL TO COORDINATE EFFECTIVELY?

IN TERMS OF EMS, IN YOUR EXPERIENCE, WHAT MAKES FOR AN IDEAL OR “BEST” PARTNER ON AN AMBJULANCE?

HOW ARE DISAGREEMENTS BETWEEN PARTNERS WORKED OUT?

WHEN YOU SEE A HIGHLY EFFECTIVE TEAM, WHAT CHARACTERISTICS MAKE THEM FUNCTION SO WELL TOGETHER?

ALTERNATIVELY, WHAT CHARACTERISTICS MAKE FOR AN INEFFECTIVE TEAM?

WHAT CAN SENIOR LEADERSHIP/MANAGEMENT DO TO PROMOTE TEAMWORK?

IS THERE ANYTHING I’VE MISSED?
